# Dental Education

**Published:** 1882-07

**Authors:** J. B. Hodgkin


### ARTICLE V.

### *Dental Education.*

BY J. B. HODGKIN, D. D. S.

The writer has no disposition to make this JOURNAL the organ of the College in which he is, unworthily perhaps, a teacher, but he feels that in view of the coming fall session of the various dental schools, he would fail to be just to his readers did he not clearly and distinctly set before them the principles of dental education by which he has striven to be guided, and the plan of teaching a careful study of the wants of the profession he represents as a journalist and teacher has led him to adopt. It cannot, he thinks, be too plainly understood by those seeking it, just what a teacher proposes to furnish in the way of a dental education, and if any are disappointed in the future, we trust it will not be from lack of clearness of exposition of the needs of dental students, as he understands them.

Wherever the writer goes, and whenever he sits down to talk with an "*elder*", in the profession, trying to gain information as to the needs of the dental profession, in answer to the question "what do dental students need," the reply is *practical training.* "Teach my son, my student, the things he would do every day." "Train him so that beyond a question he can master his work." "Drill him in the practice of dentistry." And the "*elder*" will go on to say that the study of bile pigments, white blood corpuscles, theories of Shiff, Beale, Heitzman and others, while in the highest degree interesting as theories, yet in the light of *practice* are worth little in the ordinary routine of office work. "What is best to fill teeth with," "How can I so stop this cavity as to add to the comfort of my patient longest;" "In what way can I cure this diseased root and make comfortable what is now intolerable"—these are the things that most nearly concern the patient, and, consequently most nearly concern the dentist.

And the "*elder*" will go on to tell of the rubbish of the past and beg that it be swept away. What matters it to the practitioner whether aconite gets its name from growing on rocks, or that its other name is hellebore, or whether it has three or five or two leaves. We get the tincture from the druggist all the same, and its uses and dangers are all which concern us. He will tell you how through long and weary sittings, made the more painful by alternating between vials of medicines, and making notes whilst handling these, and listening, he gained the knowledge that the diseases of children were treated differently and exhibited different symptoms from those of grown up folks.

We must say with all the emphasis we can put into words, that the complex course on physiology of that part of dentistry which involves the manufacture of artificial teeth and much else which still holds its place in the curriculum of dental teaching, is a burden too great to be borne, and that it is well nigh useless is seen in the fact that when dental students become dental graduates they rapidly lose this stock of information. They are *informed* that these things are so—they do not know them for themselves. But of the things which really concern them, and of which they cannot have too much, of this they hold on to, and add to and eclipse the teacher, because it is the legitimate study of their chosen profession.

We are no pessimist, but we cannot but feel now, even as we saw when a college student, that much of that which is taught as knowledge is but clever speculation, that ingenious hypothesis takes the place of fact, and that the love of appearing learned behind men's eyes to the requirements of the times. Dental students want learn dentistry, and it is a small matter to them whether knee joint injuries are, special dangerous, or how many successful amputations of the head of the femur have been performed. Knowledge is good· No one loves it more than the writer. He would love to spend his life in its pursuit. But realizes that life is brief, sadly brief, that much of so-called knowledge taught dental students, aye, and medical students as well, concerns them as little as the question of the moons of Mars, and is of as little value. If medical students are to be savers of lives and easers of pain it will not be out of the multitude of text books and "notes of lectures," not by trotting at the heels of the ward surgeon, or staring at a speculum the clinical professor has inserted into a diseased organ, but by seeing for themselves at short range and trying for themselves the things they need to learn. And equally so of dentistry, and it is this which must make the crown of glory of dentistry—*actual practice.* What, then, is the special want of the dental student? It is *practical knowledge;* and the writer feels sometimes, in witnessing the vague seeking after hazy truths, if truths they are, seen in schools of all sorts, that a good old-fashioned course of study in a laboratory and by the chair of a careful operator would be a better *post-graduate course* than following with a medical course the study of dentistry.

Nothing has cost the writer more labor, and some pain too, then the elimination from his course of teaching the error in the guise of truth which inevitably gathers in the teachers' mind and port-folio. But he has conscientously striven to teach nothing but the tried and tested, and not to waste the time of the student with either dead theories or valueless issues. If dentists and dental teachers would in a common-sense fashion work out these problems we feel sure it would be for the good of the profession. We would not abolish the chair of Anatomy and Physiology, (though we would banish forever Materia Medica) but we would trim these down to close facts and deductions, and we would make *dental practice* loom up so as to be more than all the rest.

The writer should add that he alone is responsible for the views thus expressed. He trusts they may be none the less carefully weighed because they are individual. There are men, honest and sincere, and seeking the good of dentistry, who are strongly advocating the blending dental schools with medical. We feel that such men have not weighed the situation justly. Dentists are already taught too much *outside of dentistry;* and a dentist need no more be a doctor of medicine to successfully pursue his chosen profession than the M. D. would need excavators and pluggers to cure infantile diseases.

